# NeSyDPP-4: discovering DPP-4 inhibitors for diabetes treatment with a neuro-symbolic AI approach

**DOI:** 10.3389/fbinf.2025.1603133

**Published:** 2025-07-21

**Authors:** Delower Hossain, Ehsan Saghapour, Jake Y. Chen

**Affiliations:** ^1^ Department of Computer Science, The University of Alabama at Birmingham, Birmingham, AL, United States; ^2^ System Pharmacology and AI Research Center (SPARC), The University of Alabama at Birmingham, Birmingham, AL, United States; ^3^ Department of Biomedical Informatics and Data Science, School of Medicine, The University of Alabama at Birmingham, Birmingham, AL, United States

**Keywords:** neuro-symbolic artificial intelligence, deep learning, DPP-4, drug discovery, machine learning, QSAR

## Abstract

**Introduction:**

Diabetes Mellitus (DM) constitutes a global epidemic and is one of the top ten leading causes of mortality (WHO, 2019), projected to rank seventh by 2030. The US National Diabetes Statistics Report (2021) states that 38.4 million Americans have diabetes. Dipeptidyl Peptidase-4 (DPP-4) is an FDA-approved target for the treatment of type 2 diabetes mellitus (T2DM). However, current DPP-4 inhibitors may cause adverse effects, including gastrointestinal issues, severe joint pain (FDA safety warning), nasopharyngitis, hypersensitivity, and nausea. Moreover, the development of novel drugs and the *in vivo* assessment of DPP-4 inhibition are both costly and often impractical. These challenges highlight the urgent need for efficient *in-silico* approaches to facilitate the discovery and optimization of safer and more effective DPP-4 inhibitors.

**Methodology:**

Quantitative Structure-Activity Relationship (QSAR) modeling is a widely used computational approach for evaluating the properties of chemical substances. In this study, we employed a Neuro-symbolic (NeSy) approach, specifically the Logic Tensor Network (LTN), to develop a DPP-4 QSAR model capable of identifying potential small-molecule inhibitors and predicting bioactivity classification. For comparison, we also implemented baseline models using Deep Neural Networks (DNNs) and Transformers. A total of 6,563 bioactivity records (SMILES-based compounds with IC_50_ values) were collected from ChEMBL, PubChem, BindingDB, and GTP. Feature sets used for model training included descriptors (CDK Extended–PaDEL), fingerprints (Morgan), chemical language model embeddings (ChemBERTa-2), LLaMa 3.2 embedding features, and physicochemical properties.

**Results:**

Among all tested configurations, the Neuro-symbolic QSAR model (NeSyDPP-4) performed best using a combination of CDK extended and Morgan fingerprints. The model achieved an accuracy of 0.9725, an F1-score of 0.9723, an ROC AUC of 0.9719, and a Matthews correlation coefficient (MCC) of 0.9446. These results outperformed the baseline DNN and Transformer models, as well as existing state-of-the-art (SOTA) methods. To further validate the robustness of the model, we conducted an external evaluation using the Drug Target Common (DTC) dataset, where NeSyDPP-4 also demonstrated strong performance, with an accuracy of 0.9579, an AUC-ROC of 0.9565, a Matthews Correlation Coefficient (MCC) of 0.9171, and an F1-score of 0.9577.

**Discussion:**

These findings suggest that the NeSyDPP-4 model not only delivered high predictive performance but also demonstrated generalizability to external datasets. This approach presents a cost-effective and reliable alternative to traditional vivo screening, offering valuable support for the identification and classification of biologically active DPP-4 inhibitors in the treatment of type 2 diabetes mellitus (T2DM).

## 1 Introduction

Diabetes mellitus (DM) is a chronic metabolic disorder characterized by elevated blood glucose levels, posing a significant global health burden. According to the World Health Organization (WHO) 2019 report, diabetes ranks among the top 10 leading causes of mortality, with an estimated 1.6 million deaths worldwide (World Health Organization: WHO, 2024; World Health Organization, 2020). In the United States, diabetes is a significant public health challenge, affecting approximately 38 million people (11.3% of the population) and leading to $327 billion in medical expenses and lost wages annually (CDC, 2024). Beyond economic costs, diabetes is associated with severe complications, including blindness, kidney failure, stroke, heart disease, and neuropathy. DM is broadly classified into type 1 diabetes mellitus (T1DM) and type 2 diabetes mellitus (T2DM), with T2DM accounting for over 90% of all cases. One decisive therapeutic target for managing Type 2 Diabetes Mellitus (T2DM) is the Dipeptidyl Peptidase-4 (DPP-4) enzyme, which plays a key role in regulating glucose metabolism. DPP-4 inhibitors, a class of FDA-approved medications, help control blood sugar levels by inhibiting this enzyme. However, current DPP-4 inhibitors have been linked to adverse effects such as gastrointestinal issues, severe joint pain, nasopharyngitis, hypersensitivity, and nausea (Huang et al., 2020). As a result, discovering safer and more effective DPP-4 inhibitors remains a critical research challenge.

Artificial intelligence (AI) has revolutionized diabetes management and drug discovery over the past two decades. Early AI models focused on predicting glucose levels, providing insulin dosage recommendations, and monitoring patients. In recent years, AI has rapidly advanced in the field of *de novo* drug design by leveraging large-scale molecular datasets. These models can not only generate novel drug candidates but also identify repurposable inhibitors and uncover complex relationships among genes, proteins, and disease mechanisms. In the field of DPP-4 inhibitor prediction, quantitative structure–activity relationship (QSAR) models have been widely developed using machine learning techniques such as random forest, support vector machines (SVMs), XGBoost, gradient boosting machines, and deep neural networks (DNNs) (Gong et al., 2021; Hermansyah et al., 2021; Ojo et al., 2021; Septiawan et al., 2022; Bustamam et al., 2021; Ajiboye et al., 2021). Although these models have demonstrated high predictive performance, they have limitations, including poor interpretability, data inefficiency, and a lack of reasoning capabilities. The black-box nature of deep learning models further complicates their use in critical healthcare applications, where transparency, logical reasoning, and explainability are vital (Hassan et al., 2022).

To address these challenges, neuro-symbolic (NeSy) AI (Hossain and Chen, 2025) has emerged as a promising paradigm that combines neural networks with symbolic reasoning for more interpretable and data-efficient learning. In contrast to traditional AI approaches that rely solely on data, Neuro-symbolic AI (NeSy AI) integrates domain knowledge with data-driven learning, enabling logical reasoning and making it especially well-suited for healthcare and drug discovery applications (Hossain et al., 2025) (Hassan et al., 2022). Studies identified numerous NeSy models that have demonstrated immense success in biomedical applications (Hossain and Chen, 2025; Yu et al., 2023; Wang W. et al., 2024), such as protein function prediction [MultiPredGO (Giri et al., 2020)], gene sequence analysis [KBANN (Towell and Shavlik, 1994)], diabetic retinopathy diagnosis [ExplainDR (Jang et al., 2021)], predicting the structure of proteins [extended KBANN (Maclin and Shavlik, 1994)], cardiotoxicity assessment hERG-LTN (Hossain et al., 2025), (Ontology) RRN (Yang et al., 2017), NSRL (Hohenecker and Lukasiewicz, 2020), Neuro-Fuzzy (Yang et al., 2020), FSKBANN (Kora et al., 2019), DeepMiRGO (Wang et al., 2019), NS-VQA (Yi et al., 2018), DFOL-VQA (Amizadeh et al., 2020), LNN (Riegel et al., 2020), NofM (Towell and Shavlik, 1991), PP-DKL (Lavin, 2022), FSD (Dobosz and Duch, 2008), CORGI (Arabshahi et al., 2021), NeurASP (Shi et al., 2019), XNMs (Teru et al., 2020), Semantic loss (Xu et al., 2018), NS-CL (Mao et al., 2019), and Logic Tensor Networks (LTNs) (Badreddine et al., 2021). In this study, we explore a hybrid neuro-symbolic approach integrating LTNs for DPP-4 bioactivity prediction, aiming to enhance predictive accuracy while enabling logical reasoning for novel drug discovery in T2DM treatment.

The paper’s main contributions are summarized as follows: 1) We developed a scalable and robust AI predictive model that demonstrates significant improvements in accuracy for predicting the potency of T2DM inhibitors. 2) A novel integration of data and rules (Knowledge) for DPP-4 inhibitor bioactivity classification. 3) We acquired and utilized a more diverse set of compound datasets, including chemical embeddings, descriptors, fingerprints, and physicochemical properties, which previous studies have not explored. The developed NeSyDPP-4 model can be used to discover novel DPP-4 active drugs by scanning large molecular datasets, such as ZINC, and identifying new candidate compounds, thereby accelerating the *de novo* design of drugs. Additionally, it facilitates QSAR model downstream applications, including virtual screening, contraindications, bioactivity indications, and other key elements of DPP-4 inhibitor therapy in clinical settings. These applications encompass docking, affinity prediction, ADMET analysis, and molecular dynamics (MD) studies for DPP-4 clinical applications.

The remainder of this manuscript is organized as follows: Section 2 provides the background, offering essential insights into the problem domain and its significance. Section 3 briefly reviews related work, highlighting existing approaches and their contributions to DPP-4 bioactivity classification. Section 4 describes the methodology, detailing the proposed approach, datasets, and algorithms used. Section 5 reports the results obtained from the experimental evaluation. Section 6 offers a discussion, interpreting and comparing the findings with existing studies. Finally, Section 7 concludes the paper with key findings and guidance for future research.

## 2 Background

### 2.1 Dipeptidyl peptidase-4 inhibitors

**FIGURE 1:**
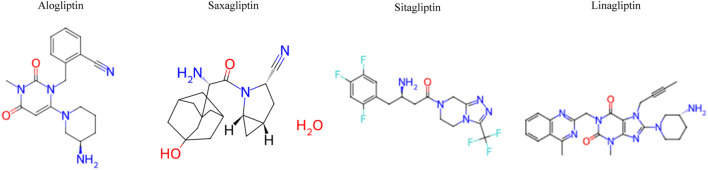
2D structure of FDA-approved dipeptidyl peptidase-4 (DPP-4) inhibitors collected from ChEMBL.

DPP-4 is an enzyme primarily involved in glucose metabolism, particularly regulating blood glucose levels in T2DM. DPP-4 is an FDA-approved target for T2DM treatment. The primary aim of the DPP-4 inhibitor is to prevent the degradation of incretin hormones and improve blood glucose control. Several FDA-approved DPP-4 inhibitors include sitagliptin, saxagliptin, linagliptin, and alogliptin (FDA, 2023; Supplementary Appendix A). In addition, Figure 1 depicts the 2D structures of FDA-approved DPP-4 inhibitors collected from ChEMBL.

### 2.2 Quantitative structure–activity relationship

QSAR modeling (Perkins et al., 2003) is a computational technique that uncovers patterns between molecular features and experimental outcomes, which helps predict the biological activity of compounds based on their chemical composition. Machine learning algorithms like artificial neural networks (ANNs), SVMs, and random forests are commonly applied to build accurate QSAR models. This approach accelerates drug discovery by enabling *in silico* assessment, reducing the reliance on extensive laboratory testing (wet-lab).

### 2.3 Symbolic AI

Good Old-Fashioned Artificial Intelligence (GOFAI), commonly referred to as Symbolic AI, is a classical AI approach emphasizing knowledge representation and reasoning. It was the dominant paradigm from the 1950s to the 1980s (Hossain and Chen, 2025), using methods like logic, rules, ontologies, decision trees, and knowledge graphs. Although symbolic AI excels in explanation, interpretability, and structured decision-making, it struggles to perform effectively at scale and with noisy data.

### 2.4 Sub-symbolic AI

Sub-symbolic AI, often called a “black box” approach, relies on large-scale data and statistical learning rather than explicit rules. Sub-symbolic (connectionist) AI has driven significant advancements in the modern AI era, such as autonomous driving systems and healthcare. For instance, a recent breakthrough contribution is building AlphaFold, a highly accurate protein structure prediction model developed by google DeepMind research. However, ANNs are the heart of this connectionist system. Although it excels in pattern recognition and handling unstructured, noisy, and big data, it lacks transparency, explainability, and reasoning.

### 2.5 Neuro-symbolic AI

Neuro-symbolic AI (Hossain and Chen, 2025) is an emerging branch of artificial intelligence constructed to bridge the gap between symbolic and connectionist approaches by integrating their strengths and eliminating their flaws. This hybrid integration aims to create AI systems that are both interpretable and capable of reasoning. Interpretable and reasoning-based AI enhances trust, transparency, and decision-making in healthcare (Giri et al., 2020; Towell and Shavlik, 1994; Jang et al., 2021; Hossain et al., 2025; Hassan et al., 2022), enabling accurate diagnoses and personalized treatments. It also improves safety, accountability, and regulatory compliance.

### 2.6 Logic Tensor Network

The LTN is a neuro-symbolic framework developed at Sony Computer Science Laboratories (Sony CSL) that enables querying, learning, and reasoning with complex data and abstract knowledge. It uses a differentiable first-order language called Real Logic to integrate logical reasoning with data-driven learning (Badreddine et al., 2021). The core advantage of this paradigm is its ability to perform reasoning, which is expressed through logical components, and is highly scalable. Additionally, it offers a comprehensive framework capable of handling supervised and unsupervised tasks, including regression, classification, and clustering. In this study, we conceived this model (LTN) for DPP-4 bioactivity classification, and more details are discussed in the *Methodology* section.

## 3 Related work

DPP-4 inhibitors are a significant class of compounds used in treating type 2 diabetes; predicting their bioactivity is essential in early drug discovery. Several machine learning-based studies have been explored for classifying DPP-4 inhibitors. For instance, QSAR models have been widely applied with descriptors such as molecular fingerprints and physicochemical properties to predict inhibitory activity. Techniques like random forests, support vector machines, and deep neural networks have shown promising results in improving classification accuracy and guiding virtual screening processes. For instance, Hermansyah et al. (2020) employed Random Forest and DNNs, achieving an accuracy of 0.9221 using CDK fingerprint and molecular properties. Bustamam et al. (2021) developed a QSAR–DNN model, yielding an accuracy of 0.904. In addition, Cai et al. (2017) applied a Naïve Bayesian (NB) approach using various fingerprint extractions, such as ECFP_4, ECFP_6, FCFP_4, and FCFP_6, reaching an accuracy of 0.872. Ulfa et al. (2021) combined Conv1D and LSTM layers for bioactivity classification, using CatBoost-selected fingerprint features, and achieved an accuracy of 0.8618. Finally, Hermansyah et al. (2023) used XGBoost with CDK and ECFP-6 features, reporting an accuracy of 0.8164. These studies highlight the progression from traditional machine learning models to advanced deep learning approaches, which have steadily improved predictive performance in DPP-4 bioactivity classification. However, to date, neuro-symbolic approaches have not been effectively integrated into DPP-4 research, even though such integration is essential for the discovery of novel chemical compounds. Moreover, previous research lacked experimentation with diverse chemical features, such as chemical language model embeddings and physicochemical properties. To address these gaps, we propose the NeSyDPP-4 strategy, which leverages Logic Tensor Networks and incorporates a wide range of feature representations, including LLaMA3.2 embeddings, PaDELPy CDKExtended fingerprints, Morgan fingerprints, chemical language model embeddings, and physicochemical properties.

## 4 Methodology

This section describes a set of procedures to determine the performance of LTNs (Badreddine et al., 2021), DNNs, and an advanced language model known as Transformer, using the ChEMBL BindingDB, PubChem, and GTP datasets related to DPP-4 inhibitors. This section covers the entire pipeline, including the material procurement, data preprocessing, feature extraction, simulation environment, network architecture, LTN knowledge-based setting, the training and inferencing phases, and the evaluation metrics used to measure the trained model’s performance.

### 4.1 Data acquisition

The study constructed a new DPP-4 cohort using four publicly available chemical compound databases, namely, ChEMBL (Gaulton et al., 2011), BindingDB (Gilson et al., 2015), PubChem, and GTP. The ChEMBL database contains more than 2 million compounds. We retrieved canonical SMILES related to the DPP-4 inhibitor with the target organism *Homo sapiens* using ID: CHEMBL284 and standard type IC_50_. The data were extracted using the ChEMBL Python API (chembl_webresource_client). The BindingDB manually uses DPP-4 string keywords (dipeptidyl peptidase-4) from their official site. Subsequently, data from PubChem in CSV format were retrieved using the following link, and GTP data were accessed via the corresponding link. In addition, to assess the model’s robustness and generalizability, we collected additional DPP-4-related data from Drug Target Common (DTC) via the provided link for external validation. Curated data can be found in the Data Availability section.

### 4.2 Data preprocessing and feature extraction

The initial bioactivity datasets comprised various irrelevant features. The curated subsets focused explicitly on the IC_50_ biological activity values, inhibitor identifiers (such as ChEMBL_ID and BindingDB_ID), and canonical SMILES representations and constrained the target organism to *Homo sapiens*. Notably, numerical IC_50_ values were reported in nanomolar (nM) units for ChEMBL, BindingDB, and GTP, while PubChem provided values in micromolar (μM) units. All IC_50_ measurements were harmonized to nanomolar units to standardize the data using the conversion formula nM = μM×1000. Subsequently, pIC_50_ values were computed from the standardized IC_50_ values through a logarithmic transformation using log10 (Equation 1) to normalize the distribution. Based on these pIC_50_ values, compounds were labeled as active or inactive according to established thresholds from prior DPP-4 chemical research (Ulfa et al., 2021). After merging all curated datasets, duplicates were removed based on the canonical SMILES column, and entries with missing values in the ID, SMILES, or IC_50_ fields were discarded. The final dataset was then split using a stratified sampling strategy with the *scikit-learn* package, followed by feature scaling using a standard scaler.

Afterward, a diverse array of features was extracted from SMILES representations, encompassing Morgan fingerprints (512, 1024, and 2048 bits), CDKExtended descriptors using PaDELPy (Yap, 2011), chemical embeddings generated via ChemBERTa-2 (Ahmad et al., 2022) and LLaMA3.2 (Ettaleb et al., 2024) from the Hugging Face model, and a comprehensive set of physicochemical properties [molecular weight, hydrophobicity-LogP, topological polar surface area (TPSA), hydrogen bond donors, hydrogen bond acceptors, and rotatable bonds] extracted using RDKit (Installation, 2024).

$${\text{pIC}}_{50}=-\log_{10}\left({\text{IC}}_{50}\times 10^{-9}\right).$$ (1)

### 4.3 LTN classification model

**FIGURE 2:**
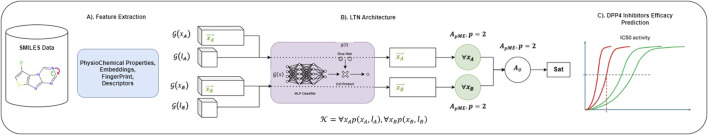
Logic Tensor Network (LTN) based Architecture of NeSyDPP4 model.

We used LTNs (Badreddine et al., 2021) to build the NeSyDPP-4 classifier. LTNs combine neural networks with first-order logic, which allows us to perform reasoning over structured knowledge while learning from data. The architecture has two key components: logic and neural networks. The visual architecture of the classification model was adopted from the LTN (shown in Figure 2). The logical mechanism contains a set of axioms or rules (explained in detail in the knowledge-based setting), and reasoning is revealed through those rules/axioms. In our context, Table 1 represents the axioms and the relevant knowledge-based component. Notably, other network configuration parameters can be found in Table 2.

**TABLE 1:** LTN knowledge-based setting for DPP-4 classification.

Content	Block
Definition of axioms	• $\forall x_{A},p\left(x_{A},l_{A}\right)$ : all the examples of class $A\left(Active=0\right)$ should have a label $l_{A}$ • $\forall x_{B},p\left(x_{B},l_{B}\right)$ : all the examples of class B (Inactive = 1) should have a label $l_{B}$
Axioms (rules and knowledge base)	$\;K=\forall x_{A}p\left(x_{A},l_{A}\right),\forall x_{B}p\left(x_{B},l_{B}\right)$
SatAgg is given by	$\;{\text{SatAgg}}_{\phi \in K}G_{\theta ,x\leftarrow D}\left(\phi \right)$
Learning and loss	$\;L=\left(1-\underset{\phi \in K}{\text{SatAgg}}G_{\theta ,x\leftarrow B}\left(\phi \right)\right)$

Note: This table was constructed inspired by the official LTN model. More detail Supplementary Appendix C.

**TABLE 2:** List of hyperparameters used for NeSyDPP4 (LTN) and DNN models for DPP-4 inhibitor classification.

Parameter	LTN	DNN
Units (input sample/hidden units)	(768,384,192,2)	(768,384,192,2)
Activation	ReLU	ReLU
No. of dense layers	3	3
Dropout	0.25	0.25
Seed	42	42
Batch size	128	128
Training epochs	100	100
Learning rate	0.00001	0.00001
Loss function	LTN pMeanError	Sparse Categorical Crossentropy
Optimizer	Adam	Adam

Note: LTN and DNN models were configured identically in terms of MLP architecture, activation function, and training setup to ensure a fair comparison.

The pMeanError aggregator, as shown in Equation 2,

$$pME\left(u_{1},\ldots ,u_{n}\right)=1-{\left(\frac{1}{n}\sum_{i=1}^{n}{\left(1-u_{i}\right)}^{p}\right)}^{\frac{1}{p}}p\geq 1,$$ (2)

Here, pMeanError is computed through universal quantification (“for all” or $\forall_{x\in A}/\forall_{x\in B}$ as shown Figure 2), which refers to the generalized mean of the deviations with respect to the truth (more detail link). Further, SatAgg’s all attributes can be defined as below (Table 1).

• SatAgg: This stands for “Satisfaction Aggregator,” an operator that aggregates the truth values of the formulas in K (if there is more than one rule).
• ϕ∈K: This part indicates that ϕ (phi) belongs to the set K. ϕ is often used to represent a predicate.
• $G\left(\theta \right)$ : This is denoted by grounding ( G ) with parameters θ. θ represents a set of parameters or weights in a model.
• x←D: D indicates the dataset of all examples (domain).
• B is a mini-batch sampled from D.

However, Figure 2 depicts an architecture composed of three segments. Segment A represents several features used to train the model, while Segment B illustrates the LTN-based classification architecture model, which was conceived from the LTN paper. Specifically, feature–label pairs $x_{A}$ and $l_{A}$ (for classes A and B, respectively) are used as input into the MLP. The output is evaluated using a universal ∀ quantification equation that influences the loss function and updates model weights via backpropagation, the same as for $x_{B}$ (all the features related to the B class). The logical formulation follows $P\left(x_{A},l_{A}\right),and\;P\left(x_{B},l_{B}\right)$ , where *p* represents the model/MLP and K denotes the complete knowledge expressed in real/first-order logic. For more details, refer to the official LTN GitHub repository.

### 4.4 Model training and validation phase

LTN, DNN, and Transformer models were trained and tested using TensorFlow 2.15.1 with Python 3.10.16 on the UAB server with an NVIDIA A100 80 GB PCIe GPU, and other dependency packages can be found in the project’s GitHub repository under environment.yml. We partitioned the data into 70:10:20 ratios over 100 epochs in the training phase. To optimize model performance, and conducted hyperparameter tuning via Grid Search (Supplementary Appendix B), with multiple training trials. The best configurations, trial 01 and trial 07, achieved the highest accuracy of 0.9726, using ReLU activation and a learning rate of 0.0001, with three layers. In addition to experimenting with the LTN, we conducted the simulation with DNNs and Transformer with Keras integrated to compare LTN performance fairly. Table 2 depicts the network configuration parameters and the simulation notebook (project GitHub), which describes the details.

**FIGURE 3:**
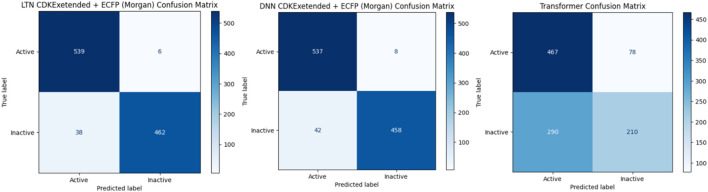
Confusion matrix of DNN, Transformer, and LTN using CDKextended + Morgan features based on DTC dataset evaluation.

Furthermore, we conducted external validation by extracting additional 1,045 data samples from the DTC dataset using the best-performing model weights. Notably, there was no duplication or overlap between the external validation data and the training dataset. The following metrics, such as accuracy, F-score (F), ROC AUC score, and Matthews correlation coefficient (MCC), were used to assess the trained model’s performance evaluation. Additionally, the confusion matrix (CM) provides a visual representation of misclassified classes (Figure 3).

Equation 3 represents the accuracy:

$$\text{Accuracy}=\frac{TP+TN}{TP+TN+FP+FN}$$ (3)

Equation 4 represents the F1-score:

$$F_{1}=\frac{2\;x\;Precision\;x\;Recall}{Precision+Recall}$$ (4)

Equation 5 represents the ROC AUC score:

$$\text{ROC AUC}=\int_{0}^{1}TPR\;d\left(FPR\right)$$ (5)

Equation 6 represents the MCC:

$$\text{MCC}=\frac{TP\times TN-FP\times FN}{\sqrt{\left(TP+FP\right)\times \left(TP+FN\right)\times \left(TN+FP\right)\times \left(TN+FN\right)}}$$ (6)

## 5 Result

This section outlines the performance of the proposed NeSyDPP-4 model in identifying potential DPP-4 inhibitors by integrating logical rules into a neural network via the LTN architecture. To achieve this, we extracted a diverse set of molecular features from each SMILES/drug representation. These include the Morgan fingerprint, CDK extended descriptors, and embeddings generated from chemical language models such as ChemBERTa-2 and LLaMA3.2 (via the Hugging Face platform). Additionally, physicochemical properties were computed using RDKit to enrich the feature space with interpretable molecular characteristics. This section presents four result grids: Table 3 shows all the features separated and combined as input results for an ablation study; Table 4 exposes the fair comparison with baseline DNN and transformer architecture performance; Table 5 summarizes the model’s performance on the external evaluation; and finally, Table 6 presents the benchmarking evaluation. To illustrate, Table 3 depicts the different input performances of NeSyDPP (LTN). The best-performing feature set is combining CDKExtended + ECFP, which yielded the highest accuracy (97.25%), F1-score (97.23%), AUC ROC (97.19%), and MCC (94.46%), while physicochemical features alone yield the lowest performance of accuracy (73.49%), F1-score (73.16%), AUC ROC (73.09%), and MCC (46.38%). ChemBERTa-2 and LLaMA3.2 performed comparably but achieved lower performance than the fingerprint-based methods. Overall, physicochemical properties alone are insufficient for effective bioactivity classification.

**TABLE 3:** NeSyDPP-4 (LTN) model’s performance comparison using various feature representations and input dimensions for DPP-4 inhibitor classification.

Model	Feature	Input	Acc	F1	AUC ROC	MCC
NeSyDPP-4 (LTN)	CDKExtended + ECFP	1024+(512 + 1024+2048)	**0.9725**	**0.9723**	**0.9719**	**0.9446**
	ECFP	1024	0.9687	0.9684	0.9680	0.9370
	ECFP	2048	0.9657	0.9654	0.9650	0.9308
	ECFP	512	0.9649	0.9646	0.9643	0.9293
	Combined All	7430	0.9634	0.9631	0.9632	0.9262
	CDKExtended	1024	0.9504	0.9499	0.9492	0.9001
	ChemBERTa-2	768	0.8956	0.8944	0.8935	0.7892
	LLaMA3.2	2048	0.8933	0.8926	0.8933	0.7854
	Physiochemical	6	0.7349	0.7316	0.7309	0.4638

Note: The table reports Accuracy (Acc), F1-score (F1), AUC-ROC, and Matthews Correlation Coefficient (MCC). The model achieved the highest performance (bold) using the combined CDKExtended and ECFP descriptors.

**TABLE 4:** Comparison of the NeSyDPP-4 (LTN) model with baseline deep learning architecture and the Transformer.

Model	Feature	Input dimension	Acc	F1	AUC ROC	MCC
NeSyDPP-4 (LTN)	CDKExtended + ECFP	1024+(512 + 1024+2048)	**0.9725**	**0.9723**	**0.9719**	**0.9446**
DNN	CDKExtended + ECFP	1024+(512 + 1024+2048)	0.9695	0.9692	0.9691	0.9385
Transformer	SMILES/Emb	212	0.7821	0.7306	0.8549	0.5641

Note: Models were trained and evaluated on identical feature sets, and results are based on internal data.

**TABLE 5:** External evaluation (DTC dataset) comparing NeSyDPP-4 with baseline DNN and Transformer model performances.

Model	Feature	Input dimension	Acc	F1	AUC ROC	MCC
NeSyDPP-4 (LTN)	CDKExtended + ECFP	1024+(512 + 1024+2048)	**0.9578**	**0.9576**	**0.9564**	**0.9171**
DNN	CDKExtended + ECFP	1024+(512 + 1024+2048)	0.9521	0.9518	0.9506	0.9057
Transformer	SMILES/Emb	212	0.6478	0.6251	0.6384	0.3095

**TABLE 6:** Comparative performance grid shows typical machine learning and deep learning models with NeSyDPP-4 for DPP-4 inhibitor classification results.

Model	Author	Metrics	Result	Reference
NeSyDPP-4	Hossain *et al*	Accuracy	**0.9578**	
Random Forest	Oky Hermansyah *et al*	Accuracy	0.9221	Hermansyah et al. (2020)
DNN	Haris Hamzah *et al*	Accuracy	0.9060	Hamzah et al. (2020)
QSAR-DNN	Alhadi Bustamam *et al*	Accuracy	0.9040	Bustamam et al. (2021)
NB	Jie Cai *et al*	Accuracy	0.8720	Cai et al. (2017)
Conv1D–LSTM	Adawiyah Ulfa *et al*	Accuracy	0.8618	Ulfa et al. (2021)
XGBoost	Oky Hermansyah *et al*	Accuracy	0.8164	Hermansyah et al. (2023)

In addition, Table 4 exhibits the internal validation results comparing the performance of NeSyDPP-4 (LTN), DNN, and Transformer models. The NeSyDPP-4 model, using CDKExtended and ECFP features, achieved the highest performance with 97.25% accuracy and an MCC of 94.46%, highlighting the strength of neuro-symbolic reasoning. The DNN model, using the same input features but without reasoning capabilities, yielded slightly lower results (96.95% accuracy and 93.85% MCC). In contrast, the Transformer model, which relied on SMILES embeddings, exhibited the weakest performance (78.21% accuracy and 56.41% MCC). These findings accentuate the effectiveness of fingerprint-based features over SMILES-based language models for bioactivity classification tasks. Furthermore, in the external evaluation, using the DTC dataset (Table 5), NeSyDPP-4 continued to outperform baseline models, confirming its strong generalization capability.

**FIGURE 4:**
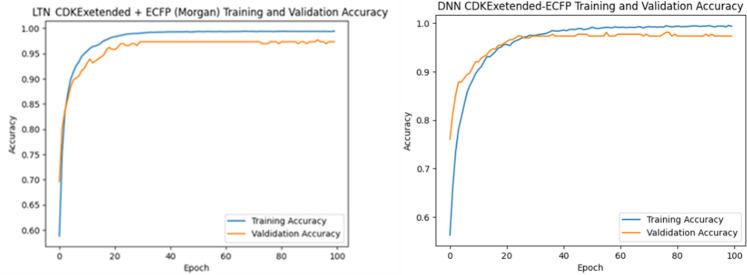
The graphs represent epoch and accuracy during the training and validation phases.

Finally, Table 6 presents a state-of-the-art (SOTA) performance comparison. The proposed NeSyDPP-4 model outperforms all baseline models across accuracy metrics. Traditional models such as Random Forest and XGBoost achieved 92.21% and 81.64% accuracy, respectively, while deep learning models like DNN and QSAR –DNN reached approximately 90%, as reported in prior benchmarks. These results underscore the superior performance of the neuro-symbolic NeSyDPP-4 model. In addition, Figure 4 illustrates the training and validation accuracy curves over 100 epochs for both the DNN and NeSyDPP-4 simulations.

## 6 Discussion

This article aimed to develop a neuro-symbolic model leveraging LTN, an integration of data and a logic-driven approach, for predicting DPP-4 inhibition in diabetes mellitus. One of the key challenges in DPP-4 inhibitor research and AI integration is the absence of unified, data and knowledge-driven experimental frameworks that support logical reasoning. Moreover, current approaches fail to effectively utilize diverse features and integrate logical rules, such as extracting embeddings from large language models (LLMs) and physicochemical properties, to predict DPP-4 bioactivity accurately. Furthermore, prior studies have focused on data-driven approaches such as DNN and Transformer. In this context, our study introduces LTN-based NeSyDPP-4, a neuro-symbolic classifier trained on the curated diverse bioactivity activity data and logical rules (Table 1), which demonstrates superior performance in predicting DPP-4 inhibitory activity compared to existing models. Notably, some studies suggest that it can be semi-interpretable since rules (Table 1) are apparently understandable by humans regarding how models should make decisions. However, the study’s findings provide valuable insights into the applicability and robustness of the LTN model, discovering bioactivity behavior. To illustrate, the utilization of this advanced machine learning technique (LTN) surpassed the state-of-the-art performance compared to other models with classification tasks; the proposed model demonstrates superior accuracy of 0.9725 and an MCC score of 0.9446 in the internal dataset for DPP-4 inhibitor bioactivity prediction. In contrast, several other studies have reported comparable results: the QSAR-DNN model by Bustamam et al. (2021) achieved an accuracy of 0.9040; Ulfa et al. (2021) reported an accuracy of 0.8618 using Conv1D–LSTM; random forest by Hermansyah et al. (2020) yielded an accuracy of 0.9221; and DNN by Hamzah et al. (2020) obtained an accuracy of 0.9060. Furthermore, the NB model by Cai et al. (2017) gained an accuracy of 0.8720, while ML-based XGBoost by Hermansyah et al. (2023) reported an accuracy of 0.8164.

**FIGURE 5:**
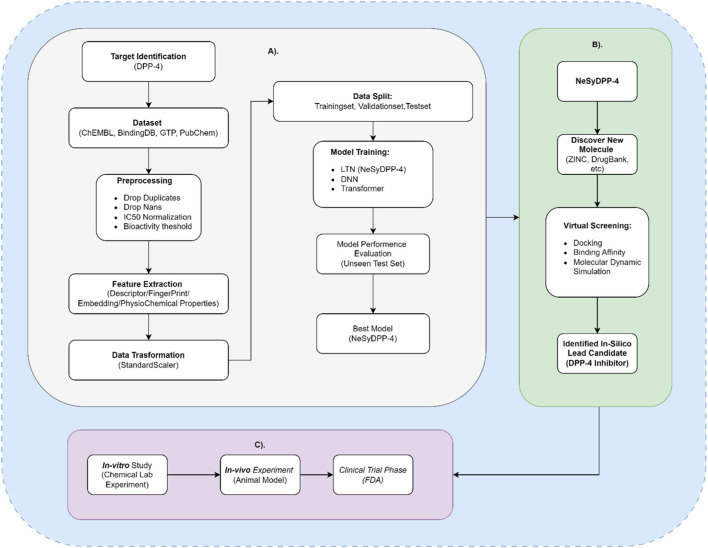
Workflow and future implications of the developed model (NeSyDPP-4). **(A)** represents a workflow from target identification to the best model (NeSyDPP-4) development; **(B)** represents the implication of the best model to discover new DPP-4 drug (FDA-approved/drug repurposing), which is known as in silico assessment; finally, based on the identified in silico compounds, *in vitro* and *in vivo* studies can be conducted in the future, as shown in **(C)**.

Overall, this study emphasizes the value of integrating neuro-symbolic modeling, which combines data-driven learning with logical rule-based reasoning, for highly accurate classification of DPP-4 inhibitor activity in the context of diabetes mellitus, a task that conventional data-driven AI approaches (DNN, Transformer) are incapable of. Significantly, the developed NeSyDPP4 model holds substantial promise for future applications, including the discovery of novel DPP-4 compounds, prediction of DPP-4-related pharmacological interactions, efficient high-throughput screening of molecular libraries to identify potential active agents, and conducting virtual screening based on identified approved/existing substance compounds that can be utilized as drug re-purposing (Figures 5B, C).

### 6.1 Limitation

Acknowledging the limitations of our study, we state that although the LTN has demonstrated significant promise, it may be incapable of incorporating external, diverse, comprehensive biological additional knowledge with neural networks due to structural limitations.

## 7 Conclusion

Diabetes mellitus is a vital global health concern, and discovering effective chemical compounds is decisive to tackling this epidemic. In this study, we develop a QSAR system to identify a therapeutic potential compound of DPP-4 inhibitors using an advanced AI framework called LTN that integrates domain-specific knowledge into neural networks. The study is a pioneer in applying the neuro-symbolic strategy in the DM domain and provides new insights that reveal a higher performance for DPP-4 bioactivity classification. The root cause of achieving such performance could be upholding learning and reasoning principles and training neural networks with rules. Furthermore, we experimented with DNN, an NLP Transformer model, whereas the LTN-based model NeSyDPP-4–QSAR attained the highest accuracy of those baselines’ approaches and prior SOTA strategies. In conclusion, the findings of this study prove that the neuro-symbolic approach for uncovering potential DPP-4 inhibitors is promising. However, an ideal direction for future work could involve integrating additional potential neuro-symbolic strategies, such as Semantic Loss and DeepProbLog, to study GLP-1, IDO, and PTP1B inhibitors, which would include extracting a variety of new descriptors and fingerprints from different datasets (PubChem and Drug Bank), focusing on regression tasks.

## Data Availability

The dataset utilized in this study can be found at: https://drive.google.com/file/d/1SGiYOyuSiirueZR3F6K0d7aHcPT43PYw/view, and the experimental code repository can be found at: https://github.com/hossain013/NeSyDPP4-QSAR.
