# Supplementary material for: NeSyDPP-4: discovering DPP-4 inhibitors for diabetes treatment with a neuro-symbolic AI approach
Source: Front Bioinform. 2025 Jul 21;5:1603133. doi: 10.3389/fbinf.2025.1603133 (PMC12319772; doi:10.3389/fbinf.2025.1603133)
Supplement: Supplementary file 1 [file DataSheet1.docx]

NeSyDPP4-QSAR: Discovering DPP-4 Inhibitors for Diabetes Treatment with a Neuro-symbolic AI Approach

**Appendix A**: A list of FDA, EU, EMA (European Medicines Agency), JAPAN, and KOREN BODY approved DPP4 inhibitors’ structure and respective 3D compound structures images as below.

| ChEMBL ID | Target | Approved Body | Smiles | References |
| --- | --- | --- | --- | --- |
| CHEMBL376359 | Alogliptin | FDA | Cn1c(=O)cc(N2CCC[C@@H](N)C2)n(Cc2ccccc2C#N)c1=O | FDA Approved DPP-4 2023, |
| CHEMBL1929396 | Anagliptin | Japan | Cc1cc2ncc(C(=O)NCC(C)(C)NCC(=O)N3CCC[C@H]3C#N)cn2n1 | FDA Approved DPP-4 2023, Wikipedia 2018 |
| CHEMBL3707235 | Gemigliptin | Korea | N[C@@H](CC(=O)N1CCc2c(nc(C(F)(F)F)nc2C(F)(F)F)C1)CN1CC(F)(F)CCC1=O | FDA Approved DPP-4 2023, Wikipedia 2018 |
| CHEMBL237500 | Linagliptin | FDA | CC#CCn1c(N2CCC[C@@H](N)C2)nc2c1c(=O)n(Cc1nc(C)c3ccccc3n1)c(=O)n2C | FDA Approved DPP-4 2023, |
| CHEMBL385517 | Saxagliptin | FDA | N#C[C@@H]1C[C@@H]2C[C@@H]2N1C(=O)[C@@H](N)C12CC3CC(CC(O)(C3)C1)C2 | FDA Approved DPP-4 2023, |
| CHEMBL1422 | Sitagliptin | FDA | N[C@@H](CC(=O)N1CCn2c(nnc2C(F)(F)F)C1)Cc1cc(F)c(F)cc1F | FDA Approved DPP-4 2023, |
| CHEMBL2147777 | Teneligliptin | Japan | Cc1cc(N2CCN([C@@H]3CN[C@H](C(=O)N4CCSC4)C3)CC2)n(-c2ccccc2)n1 | FDA Approved DPP-4 2023, Wikipedia 2018 |
| CHEMBL142703 | Vildagliptin | EMA | N#C[C@@H]1CCCN1C(=O)CNC12CC3CC(CC(O)(C3)C1)C2 | FDA Approved DPP-4 2023, Wikipedia 2018 |

| 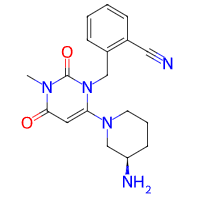 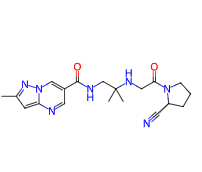 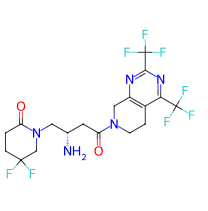 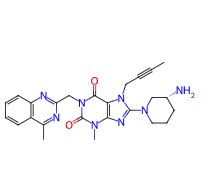  Saxagliptin  Sitagliptin  Teneligliptin  Vildagliptin  Linagliptin  Gemigliptin  Anagliptin  Alogliptin  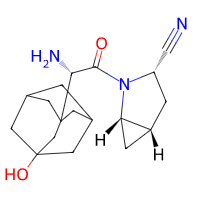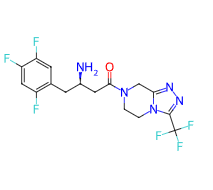 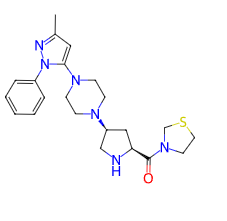 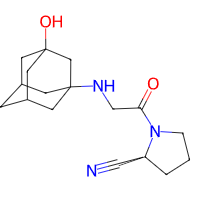  Fig. DPP-4 Inhibitors |
| --- |

More approved inhibitors can be found in ChEMBL, Drug Bank, and Wikipedia.

**Appendix B**: Hyperparameter optimization

| Table 7: Hyperparameter optimization results using Grid Search | | | | | | |
| --- | --- | --- | --- | --- | --- | --- |
| Trial | **Units 1** | **Units 2** | **Units 3** | **Activation** | **Learning Rate** | **Acc Score** |
| 01 | **768** | **384** | **192** | **relu** | **0.0001** | **0.9726** |
| 07 | 768 | 384 | 96 | relu | 0.0001 | 0.9726 |
| 08 | 768 | 384 | 96 | relu | 0.001 | 0.9695 |
| 00 | 768 | 384 | 192 | relu | 1e-05 | 0.9680 |
| 02 | 768 | 384 | 192 | relu | 0.001 | 0.9665 |
| 06 | 768 | 384 | 96 | relu | 1e-05 | 0.9627 |
| 03 | 768 | 384 | 192 | elu | 1e-05 | 0.9604 |
| 04 | 768 | 384 | 192 | elu | 0.0001 | 0.9604 |
| 09 | 768 | 384 | 96 | elu | 1e-05 | 0.9566 |
| 05 | 768 | 384 | 192 | elu | 0.001 | 0.4539 |
|  | | | | | | |

**Appendix C**: LTN / Knowledge-based Setting

The construction of all the axioms components was conceived from the official LTN GitHub framework (Badreddine et al., 2021)

Classification:

- *Domains*
  - $items$, denoting the examples from the DPP-4 dataset
  - $labels$, representing the class labels (Based on IC50 values active inactive classes)
- *Define Variables*
  - $\begin{aligned} x_{active},x_{inactive}, \end{aligned}$ , for the positive examples of classes $A and B$
  - $x$for all examples
  - $\begin{aligned} D\left( x_{A} \right)=D\left( x_{B} \right)=D\left( x \right)=items \end{aligned}$
- *Define Constants*
  - $\begin{aligned} L_{active},L_{inactive} \end{aligned}$the labels of classes $\begin{aligned} A\left( 0 \right) and B\left( 1 \right) \end{aligned}$ Respectively.
  - $\begin{aligned} D\left( l_{A} \right)=D\left( l_{B} \right)=labels \end{aligned}$ (active inactive pic50 based)
- *Define the P predicate.*
- $\begin{aligned} \rho\left( x,l \right) \end{aligned}$ Denoting the fact that item $x$ is classified as $l$;
- $\begin{aligned} D_{in}\left( P \right)=items,labels \end{aligned}$.
- *Connectives:*
- *For All* $\boldsymbol{\forall,}$ *And* $\boldsymbol{\wedge,}$ *Not* $\neg,$*Or* $\vee$, *Implies* $\begin{aligned} \Longrightarrow\end{aligned}$

Note: our case only And $\boldsymbol{\wedge}$

- *Axiom*
  - $\forall x_{A},p\left( x_{A},l_{A} \right)$: all the examples of class $A\left( active \right)$should have a label $l_{A}$
  - $\forall x_{B}, p\left( x_{B},x_{B} \right):$all the examples of class $B$ $\left( Inactive \right)$ should have a label$l_{B}$

Notice that rules about exclusiveness, such as $\forall\left( P\left( x,l_{A} \right)\Longrightarrow\left( \neg P\left( x,l_{B} \right)\boldsymbol{\wedge,} \neg P\left( x,l_{C} \right) \right) \right)$ They are omitted since such constraints are already imposed by the grounding of $P\mathbf{,}$ below, more specifically by the softmax function.

- Grounding:
  - - - $\mathcal{G}\left( \mathrm{items} \right)=R^{N}$, items are described by $N$ features:
      - $\mathcal{G}\left( \mathrm{labels} \right)=N^{2},$We use an encoding to represent classes.
      - $\mathcal{G}\left( x_{\mathrm{active}} \right)\in R^{m_{\mathbb{1}}\times N}$, that is, $\mathcal{G}\left( x_{active} \right)$ is a sequence of $m_{1}$ examples of class$\mathrm{active}$;
      - $\mathcal{G}\left( x_{\mathrm{inactive}} \right)\in R^{m_{\mathbb{2}}\times N}$, that is, $\mathcal{G}\left( x_{\mathrm{inactive}} \right)$ is a sequence of $m_{2}$ examples of class$\mathrm{inactive}$;
      - $\mathcal{G}\left( x \right)\in R^{\left( m_{\mathbb{1}}+m_{\mathbb{2}} \right)\times N},$ that is, $\mathcal{G}\left( x \right)$ It is a sequence of all the examples.
      - $\mathcal{G}\left( l_{A} \right)=0,\mathcal{G}\left( l_{B} \right)=1$;
      - $\mathcal{G}\left( P \mid\theta\right):x,l\mapsto l^{\top}\cdot softmax\left( \mathrm{MLP}_{\theta}\left( x \right) \right),$where $MLP$has two output neurons corresponding to as many classes, notably in our cases, two classes as we explained early, and $\cdot$ denotes the dot product as a way of selecting an output for $\mathcal{G}\left( P \mid\theta\right)$. Multiplying the $MLP$ output by the probability. $l^{\top}$ Gives the probability corresponding to the class denoted by$l$.
